# Highly Purified Conjugates of Natural Chlorin with Cobalt Bis(dicarbollide) Nanoclusters for PDT and BNCT Therapy of Cancer

**DOI:** 10.3390/bioengineering9010005

**Published:** 2021-12-25

**Authors:** Maria K. Fedotova, Maksim N. Usachev, Ekaterina V. Bogdanova, Ekaterina Diachkova, Yuriy Vasil’ev, Vladimir I. Bregadze, Andrey F. Mironov, Mikhail A. Grin

**Affiliations:** 1 Department of Chemistry and Technology of Biologically Active Compounds, Medicinal and Organic Chemistry, Institute of Fine Chemical Technologies, MIREA-Russian Technological University, 86 Vernadsky Avenue, 119571 Moscow, Russia; mk.khrenova@gmail.com (M.K.F.); maximus021989@mail.ru (M.N.U.); mironov@mitht.ru (A.F.M.); michael_grin@mail.ru (M.A.G.); 2 A.N. Nesmeyanov Institute of Organoelement Compounds, Russian Academy of Sciences, 28 Vavilov Str., 119991 Moscow, Russia; bogdanovakatte@mail.ru (E.V.B.); bre@ineos.ac.ru (V.I.B.); 3 Department of Oral Surgery, Borovsky Institute of Dentistry, I.M. Sechenov First Moscow State Medical University (Sechenov University), Trubetskaya St. bldg. 8\2, 119435 Moscow, Russia; 4 Department of Fundamental Medical Disciplines, Medical Faculty, Moscow Region State University (MRSU), Str. Radio 10 Build 1, 105005 Moscow, Russia; 5 Department of Operative Surgery and Topographic Anatomy, I.M. Sechenov First Moscow State Medical University (Sechenov University), Trubetskaya St. bldg. 8\2, 119435 Moscow, Russia; y_vasiliev@list.ru; 6 Department of Prosthetic Dentistry, Dental Faculty, Kazan State Medical University of the Ministry of Health of Russia, Str. Butlerova 49, 420012 Kazan, Russia

**Keywords:** anticancer, boron neutron capture therapy, nanoconjugate, neutron sensitizer, photodynamic therapy, photosensitizer, theranostic, UHPLC-HR MS/MS, preparative chromatography

## Abstract

To combine the neutron-capturing and photodynamic properties of boron nanoclusters and derivatives of natural chlorins, respectively, in one molecule, conjugate of chlorin e6 methyl ester with cyclen and dioxane and nitrile derivatives of cobalt bis(dicarbollide) were synthesized. The conditions for the purification of compounds by HPLC were selected since the work with natural compounds is complicated by the production of closely related impurities.

## 1. Introduction

Currently, the efforts of researchers working in the field of oncology are aimed at combining various methods of influencing tumors. Boron neutron capture therapy (BNCT) is one of the most promising methods for treating head and neck tumors [1,2]. This is a binary non-invasive method based on the nuclear reaction of two non-toxic agents−non-radioactive isotopes ^10^B and low-energy thermal neutrons [3,4,5,6,7]. The effectiveness of BNCT is ensured by a high concentration of 10B atoms in a cancer cell; however, boron clusters themselves do not possess targeting properties. For targeted delivery and the tracking of tumor accumulation, it is promising to use conjugates of boron clusters with photosensitizers (PS), which can be derivatives of natural chlorins [8,9,10]. Due to their ability to absorb light in the near infrared region of the spectrum (650–700 nm), chlorin derivatives are widely used in photodynamic therapy (PDT) and fluorescence diagnostics (FD) [11,12]. The combination of the properties of a photosensitizer and a BNCT agent in one conjugate makes the latter a theranostic capable of visualizing a tumor and realizing a binary therapeutic effect on it. In this work, we study the conditions for the addition of boron clusters to the secondary nitrogen atoms of cyclen in the chlorin–cyclen conjugate. We previously described the synthesis of this conjugate and its properties as a chelating agent for transition metals [13,14].

In this article, the problem of obtaining a chlorin–cyclen conjugate with several fragments of bis(1,2-dicarbollide) cobalt is solved in order to increase the number of boron atoms in the PS molecule, and, as a consequence, increase the efficiency of BNCT. Since the cyclen molecule in the conjugate has three nucleophilic centers, the reactions with bis(1,2-dicarbollide) cobalt derivatives are ambiguous and there is a high probability of the formation of mono-, di- or tri-substituted products.

The preparation of conjugates of natural chlorins with boron clusters is accompanied by an abundance of reaction by-products. In this regard, the issue of the identification and isolation of target compounds in an individual state from the reaction mixture is relevant. Modern approaches to the identification of natural porphyrins and their derivatives are increasingly based on the use of high-performance liquid chromatography with high-resolution tandem mass spectrometry [15,16]. This method is also actively used in the analysis of porphyrins in various biological samples [17,18]. Typically, the highly efficient selective separation of reaction products is based on the use of modern preparative chromatographic systems such as “ÄKTA Pure” [19,20]. In this work, the high potential of using preparative and analytical liquid chromatography methods to obtain high-purity conjugates with boron clusters is demonstrated. The identity of the conjugates of natural chlorin with cobalt bis(dicarbollide) nanoclusters was confirmed by HRMS (ESI) mass spectrometry by the presence of the pseudo-molecular ion peaks [M + H]^+^, [M + 2H]^2+^, [M-H]- (5 ppm deviation with respect to calculated mass) and characteristic fragment ions, produced in a HCD collision energy cell. The purification of conjugates was performed by reversed phase chromatography using the preparative chromatographic system “ÄKTA Pure 25”.

## 2. Materials and Methods

**Compound 2.** A 5-fold excess of the nitrile derivative of bis(1,2-dicarbollide) cobalt was added to the chlorin–cyclen conjugate **1**. The reaction proceeded by refluxing in acetonitrile under an inert argon atmosphere for 8 h. The progress of the reaction was monitored by an increase in the chromatographic mobility of the product and a shift in the maximum absorption peak of the reaction mixture from 664 nm to 642 nm.

UV (λ max/nm): 400, 500, 610, 662. 

HRMS (ESI): *m*/*z* [M + H]^+^ = 1157.7794 [M + H]^2+^ = 579.3929.

**Compounds 3 and 4.** A 5-fold excess of dioxane derivative of bis(1,2-dicarbollide) cobalt was added to chlorin–cyclen conjugate **1**. The reaction proceeded by refluxing in acetonitrile under an inert argon atmosphere for 6 h. The progress of the reaction was monitored by an increase in the chromatographic mobility of the product and a shift in the maximum absorption peak of the reaction mixture from 664 nm to 642 nm.

**(3)** UV (λ max/nm): 400, 500, 610, 663. 

HRMS (ESI): *m*/*z* [M + H]^+^ = 1190.7883 [M + H]^2+^ = 595.8962. 

**(4)** UV (λ max/nm): 400, 500, 610, 663. 

HRMS (ESI): *m*/*z* [M + H]^+^ = 1602.1148.

Purification of the target compounds was performed using the preparative chromatographic system “ÄKTA Pure 25”, including a binary pump of mobile phase with a high-pressure gradient, a sample injector with a 0.5 mL loop, a UV detector at 220 nm, an automatic fraction collector (General Electric Healthcare, Chicago, IL, USA). Chromatographic separation was performed on a preparative column “Biotage Snap Discovery C18” 120 mm × 25 mm, 10 μm particle size (“Biotage”, Charlotte, NC, USA). The solvents consisted of 0.1% formic acid (HPLC grade “Fluka”, USA, cat. no. 56302-1L,) in Milli-Q water (18.2 cm-1) (eluent A) and isopropyl alcohol (HPLC grade “Scharlau”, Barcelona, Spain, catalog number 603-117-00-0) (eluent B). Gradient started at 95% A (held for 1 min), was decreased to 5% A within 15 min (held for 5 min). The flow rate was 25 mL/min.

Identification of the target compounds was performed at the ultra-high performance liquid chromatographic system “Vanquish” coupled with a hybrid high-resolution mass spectrometer “Q-Exactive HF-X” (Thermo Fisher Scientific, Waltham, MA, USA). For the analysis compounds, a reversed-phase analytical column “Pyramid” 75 × 2, 1.8 μm particle size (MACHEREY-NAGEL, Düren, Germany) was used. The solvents consisted of 0.1% formic acid (HPLC grade “Fluka”, USA, cat. no. 56302-1L) in Milli-Q water (18.2 cm^−1^) (eluent A) and isopropyl alcohol (HPLC grade “Scharlau, Spain, catalog number 603-117-00-0) (eluent B). Gradient started at 95% A (held for 1 min) and was decreased to 5% A within 10 min (held for 2 min). The flow rate was 0.3 μL/min. The high-resolution mass spectrometer was equipped with a HESI-II ion source and nitrogen from a N_2_ generator (Genius Scotland) as gas for the ion source and the higher energy collisional dissociation (HCD) experiments. Sheath gas—45 a.u.; aux gas—35 a.u.; sweet gas—2 a.u. Spray voltage 4.1 kV. The aux gas heater temperature was 200 °C. The mass spectrometer was operated in positive and negative ionization modes. Full scan analysis from *m*/*z* 350 to 2200 with a resolution of 70,000 (FWHM) was performed in all experiments. For the analysis of the target compounds and elucidation of impurities in reaction mixture, Parallel Reaction Monitoring (PRM) was performed by adding an inclusion list of observed *m*/*z*. The resolution for all MS/MS experiments was set to 17,500 (FWHM) by stepped NCE 35, 40, and 45 eV.3.

## 3. Results and Discussion

**Scheme 1:**
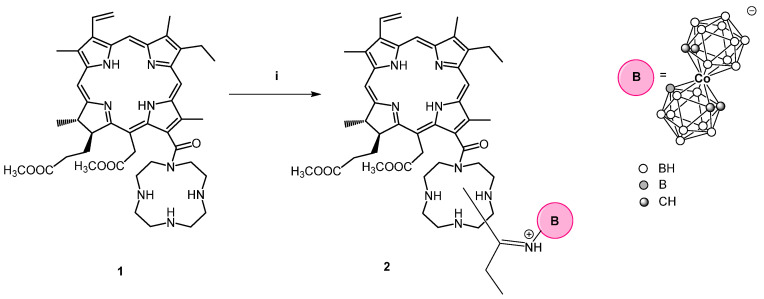
Reagents and conditions: **i**, [8-EtC≡N-3,3′-Co(1,2-C_2_B_9_H_10_)(1′,2′-C_2_B_9_H_11_)], CH_3_CN, t = 81 °C, Ar.

At the first stage of the work, the reaction of a chlorin–cyclen conjugate with a nitrile derivative of bis(1,2-dicarbollide) cobalt was realized (Scheme 1). The latter was obtained in the scientific group of Professor V.I. Bregadze, and studied its modification by various nucleophiles [21]. In this work, the remainder of the cyclen molecule was used as the nucleophile. The result turned out to be unexpected, as in the course of this reaction, only one boron cluster was added, which, apparently, can be associated with the different steric accessibility of secondary nitrogen atoms in the cyclen, as well as the fixed position of the boron cluster linked by a short and hard spacer.

**Figure 1:**
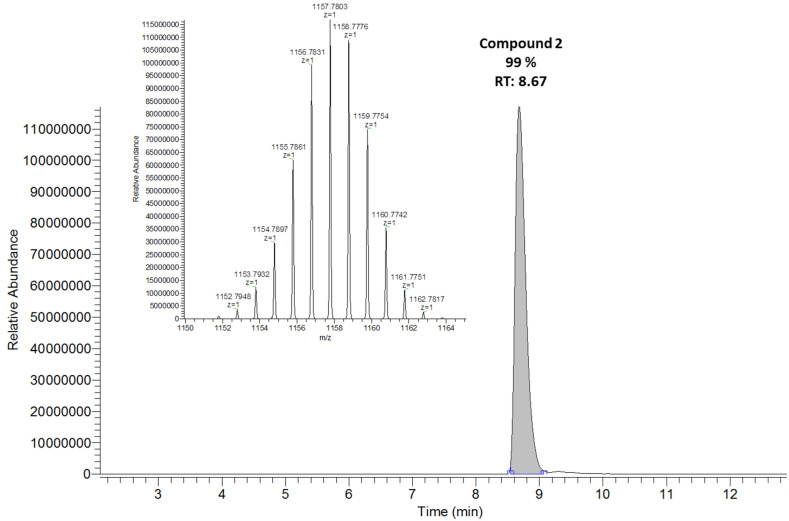
Total ion chromatogram of collected fraction by purifying reaction mixture by ÄKTA Pure 25. Percentage of compound **2** = 99%, RT = 8.67 min, *m*/*z* [M + H]^+^ = 1157.7794.

The reaction mixture was measured by LC-HRMS (Figure 1), according to which the reaction mixture contained 27% of the target conjugate **2**. Apparently, such a low yield of this reaction was due to the presence of unreacted starting materials in the reaction products due to the steric complexity of the reaction. The purification of the reaction mixture was performed by “ÄKTA Pure 25”, and compound **2** was collected from 8.54 to 9.04 min.

The structure of the resulting conjugate was confirmed by high-resolution mass spectrometry (Figure 1), where a molecular ion of compound **2** was detected (*m*/*z* [M + H]^+^ = 1157.7794; [M + H]^2+^ = 579.3929) with a set of signals corresponding to the isotopic composition of cobalt, which corresponded to the calculated data. As well as characteristic signals of protons of the chlorin macrocycle and signals of boron atoms, characteristics of the bis(1,2-dicarbollide) backbone were found by ^1^H and ^11^B NMR spectroscopy. 

**Scheme 2:**
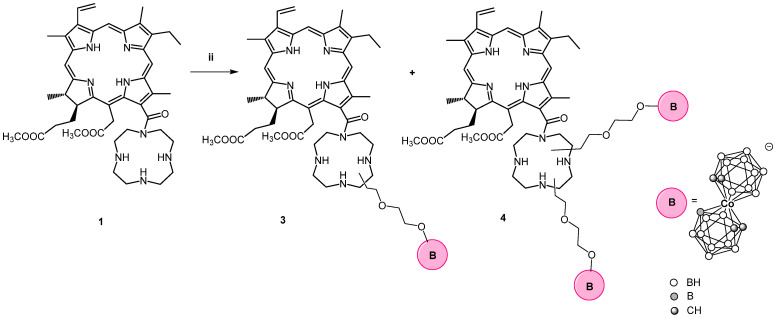
Reagents and conditions: **ii**, [8-O(CH_2_CH_2_)_2_O-3,3′-Co(1,2-C_2_B_9_H_10_)(1′, 2′-C_2_B_9_H_11_)], CH_3_CN, t = 81 °C, Ar.

Another scheme for the preparation of the boron-containing chlorin included the interaction of the previously described chlorin–cyclen **1** with a dioxonium derivative of bis(1,2-dicarbollide) cobalt. Earlier, our research group studied the reaction of the nucleophilic opening of the dioxonium ring with aminoamides of natural chlorins [8]. This approach was extended to the cyclic chlorin derivative (Scheme 2), while the question of the number of attached clusters remained open. In the course of the reaction, a mixture of conjugates was obtained, the subsequent separation of which by HPLC and the determination of the components of the mixture showed the presence of mono- and di-substituted derivatives.

**Figure 2:**
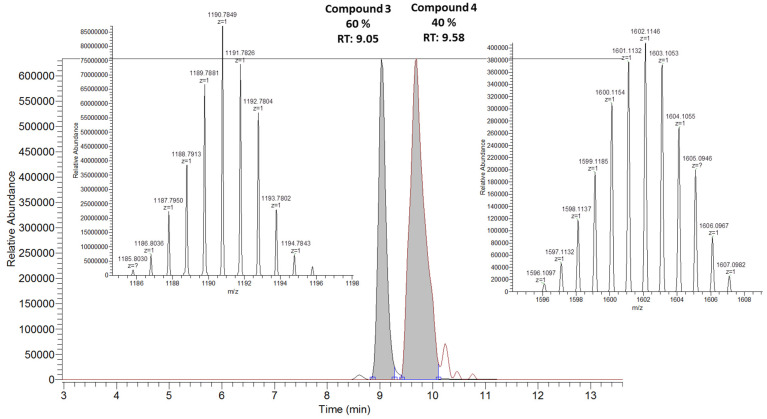
Total ion chromatogram of mixture of compound **3** (relative abundance = 60%, RT = 9.05 min, *m*/*z* [M + H]^+^ = 1190.7849) and compound **4** (relative abundance = 40%, RT = 9.58 min, *m*/*z* [M + H]^+^ = 1602.1146).

The reaction mixture was measured by LC-HRMS, according to which the reaction mixture contained 60% of the mono-substitution conjugate **3** and 40% of the di-substitution conjugate **4**. The purification of the reaction mixture was performed by “ÄKTA Pure 25”. High-resolution mass spectra were taken from the purified compounds, in which the characteristic peaks of molecular ions corresponding to mono- (*m*/*z* [M + H]^+^ = 1190.7849) and di-substitution (*m*/*z* [M + H]^+^ = 1602.1146) conjugates were found (Figure 2). Each characteristic peak contained a set of signals corresponding to the isotopic composition of cobalt, which corresponded to the calculated data. Additionally, characteristic signals of the protons of the chlorin macrocycle and signals of boron atoms characteristic of the bis(1,2-dicarbollide) backbone were found by ^1^H and ^11^B NMR spectroscopy, both in compound **3** and **4**.

## 4. Conclusions

In this work, mono- and di-boron-substituted chlorins were obtained. Moreover, in the case of nitrile derivatives, only one product was formed. At the same time, when using a dioxonium derivative, it was possible to introduce a second boron cluster. Apparently, this was due to steric effects and the length of the spacer group between the macrocycle and boron polyhedron. The conditions for the chromatographic purification of the obtained compounds were selected, which made it possible to obtain individual substances. In the future, it is planned to conduct biological tests of the obtained clusters to assess their accumulation in cells and the effectiveness of their use in PDT and BNCT.

## Data Availability

Data available on request due to restrictions eg privacy or ethical. The data presented in this study are available on request from the corresponding author. The data are not publicly available due to Funding requirements.
